# Perioperative dynamics of the prognostic nutritional index predict recurrence and survival after resection for colorectal liver metastases

**DOI:** 10.1097/MD.0000000000049748

**Published:** 2026-07-17

**Authors:** Kyota Tatsuta, Mayu Sakata, Yuya Iwase, Toru Takagi, Akio Matsumoto, Shinya Ida, Kosuke Sugiyama, Tadahiro Kojima, Toshiya Akai, Kakeru Torii, Satoru Furuhashi, Mikihiro Shimizu, Makoto Takeda, Yoshifumi Morita, Hirotoshi Kikuchi, Yoshihiro Hiramatsu, Kiyotaka Kurachi, Hiroya Takeuchi

**Affiliations:** a Department of Surgery, Hamamatsu University School of Medicine, Hamamatsu, Shizuoka, Japan; b Center for Clinical Research, Hamamatsu University School of Medicine, Hamamatsu, Shizuoka, Japan; c Division of Surgical Care, Morimachi, Hamamatsu University School of Medicine, Hamamatsu, Shizuoka, Japan; d Department of Perioperative Functioning Care and Support, Hamamatsu University School of Medicine, Hamamatsu, Shizuoka, Japan.

**Keywords:** colorectal cancer, liver metastases, prognostic nutritional index, recurrence, survival

## Abstract

We evaluated how perioperative changes in the prognostic nutritional index (PNI) influence recurrence and survival after hepatectomy for colorectal cancer liver metastases (CRLM). We conducted a retrospective analysis of 108 patients who underwent hepatectomy for CRLM. Preoperative and postoperative PNI levels were compared, and patients were categorized into high (postoperative PNI equal to or higher than preoperative PNI) and low (postoperative PNI lower than preoperative PNI) groups. Statistical analyses included the Kaplan–Meier analysis, log-rank tests, Cox proportional hazards regression models, and Akaike information criterion. The median observation period was 3.3 years. The Kaplan–Meier analysis showed worse overall survival and recurrence-free survival in the low group. Subgroup analysis demonstrated reproducibility regardless of patient background, era of surgery, surgical outcomes, neoadjuvant or adjuvant chemotherapy, and liver metastasis status. Multivariate analysis identified low group assignment and synchronous liver metastasis as independent predictors of recurrence (hazard ratio [HR], 3.788; 95% confidence interval [CI], 2.030–7.621; HR, 3.338; 95% CI, 1.715–6.964, respectively) and survival (HR, 4.484; 95% CI, 1.945–10.334; HR, 2.529; 95% CI, 1.183–5.407, respectively). The Akaike information criterion analysis showed that perioperative shifts in PNI were superior to preoperative or postoperative PNI alone in predicting outcomes. Perioperative shifts in PNI predicted recurrence and prognosis post-hepatectomy for CRLM more accurately compared to using preoperative or postoperative PNI alone.

## 1. Introduction

Worldwide, colorectal cancer accounts for 1.4 million new cases and over 600,000 deaths annually, with two-thirds of these cases attributed to liver metastases.^[1]^ Hepatectomy is the only treatment that can potentially extend survival or even cure patients with colorectal cancer liver metastases (CRLM). Approximately 80% of patients with CRLM have an unresectable diagnosis at the time of discovery. However, recent innovations in the treatment of CRLM, including chemotherapy, expanded surgical criteria, and novel surgical techniques, have enabled hepatectomies in such patients.^[2]^ Despite these developments, the prognosis remains poor, with a 5-year survival rate of 46% for resectable liver metastases and 32% for cases converted from initially unresectable to resectable.^[3]^

Many clinical prediction models have been developed.^[4,5]^ These models were often calculated using preoperative clinicopathological variables, such as the size and number of liver metastases. Additionally, preoperative nutritional status predicts recurrence and prognosis after hepatectomy for CRLM.^[6]^ Recently, the European Society for Parenteral and Enteral Nutrition (ESPEN) expert group highlighted the perioperative nutritional and metabolic management of surgical patients.^[7]^ Recent studies have examined the effect of perioperative nutritional changes on the recurrence and prognosis of various cancer types.^[8–10]^ They used the prognostic nutritional index (PNI),^[11]^ calculated from serum albumin levels and total lymphocyte counts, to assess perioperative nutritional status. Groups with postoperative PNI levels higher than preoperative PNI based on study-specific cutoff values reported significantly better prognoses of various cancer types.^[8,9]^ However, whether perioperative shifts in PNI are potent predictors of recurrence and prognosis after hepatectomy for CRLM is unknown. We hypothesized that increasing postoperative PNI levels after hepatectomy for CRLM would improve patient prognosis. This study focused on determining whether perioperative shifts in PNI affect outcomes after hepatectomy for CRLM.

## 2. Methods

### 2.1. Study design and patient population

The Institutional Review Board of Hamamatsu University School of Medicine approved this study (approval number: 17-132), which was carried out in accordance with the ethical principles of the Declaration of Helsinki (1964) and its subsequent amendments or equivalent guidelines. Opt-out-based informed consent was obtained from the patients. Between January 2001 and December 2023, 147 patients who underwent hepatectomy for CRLM at the Hamamatsu University School of Medicine were retrospectively reviewed. The pathological stage was diagnosed based on the Union for International Cancer Control TNM classification of malignant tumors (8th edition). The inclusion criteria for this study were as follows: age over 20 years; ECOG performance status of 0 or 1; absence of synchronous malignancies; and postoperative survival of >30 days. Patients were deemed ineligible based on the following exclusion criteria: incomplete data (n = 35), simultaneous pulmonary metastasis (n = 2), and the liver-1st approach for synchronous metastases (n = 2). Overall, 108 patients were included in this study.

### 2.2. Definition of perioperative PNI levels

**Figure 1.**
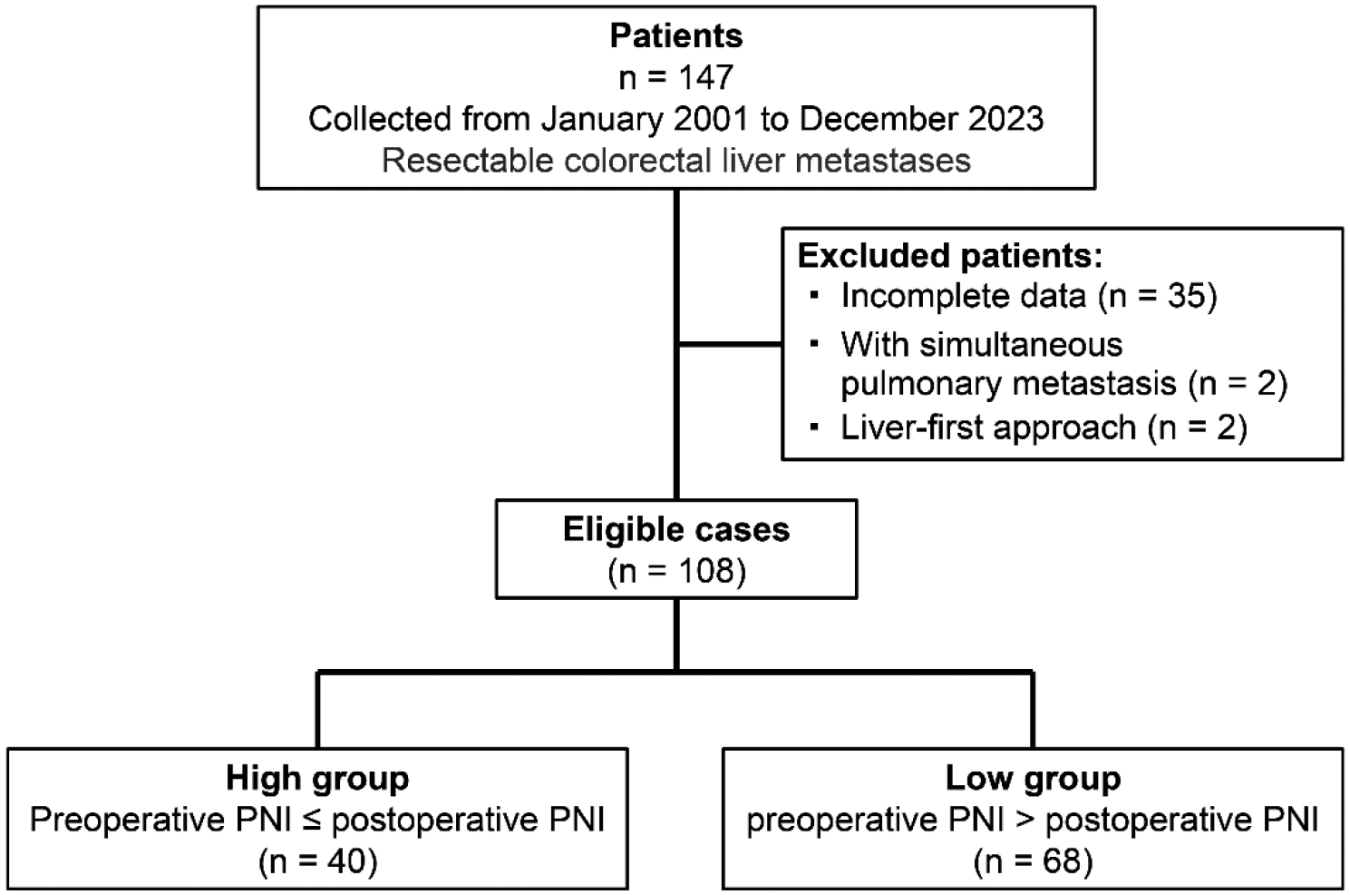
Flow diagram of the presented study. PNI = prognostic nutritional index.

The PNI was calculated as 10 × serum albumin (g/dL) + 0.005 × total lymphocyte count (per mm^3^).^[11]^ Preoperative PNI was calculated from the results of laboratory tests performed within 4 weeks before surgery for CRLM; if multiple tests were performed, the best result was adopted. Postoperative PNI was calculated from the results of laboratory tests performed approximately 4 weeks (3–7 weeks) after surgery. The same calculation was performed when the patient remained hospitalized for the treatment of postoperative complications at 4 weeks. These calculations were performed following previously reported methods with some modifications.^[10]^ Comparing preoperative PNI and postoperative PNI, we categorized the patients into 2 groups: the high group (postoperative PNI equal to or higher than preoperative PNI, n = 40) and the low group (postoperative PNI lower than preoperative PNI, n = 68) (Fig. 1).

### 2.3. Treatment and postoperative complications

All surgeries complied with the clinical practice guidelines established by the Japanese Society for Cancer of the Colon and Rectum.^[12,13]^ All patients without unresectable extrahepatic tumors underwent hepatic resection if curative resection left a sufficient remnant liver, regardless of the size, number, or location of the liver metastases. A parenchymal-sparing approach is generally adopted during hepatectomy. Anatomical resections were determined according to the Brisbane 2000 nomenclature of liver anatomy,^[14]^ while nonanatomical resections were classified as limited partial resections. Major hepatectomy was defined as the resection of 3 or more consecutive Couinaud hepatic segments. Neoadjuvant chemotherapy was administered for borderline resectable or initially unresectable liver metastases. Postoperative complications were assessed using the Clavien–Dindo classification, with grade 2 or higher events considered postoperative complications.^[15]^

### 2.4. Adjuvant therapy and follow-up

The decision to administer postoperative adjuvant chemotherapy was left to the discretion of the attending physician owing to insufficient evidence in the Japanese Society for Cancer of the Colon and Rectum guidelines.^[12,13]^ Patients were followed up every 3 months for the 1st 3 years, and every 6 months thereafter. At each visit, blood tests were conducted to evaluate tumor markers, and chest, abdominal, and pelvic computed tomography (CT) scans were performed every 6 months. Recurrence after CRLM was defined as the detection of new local or distant metastatic lesions on CT, magnetic resonance imaging, or positron emission tomography–CT scans. Management of recurrent tumors included resection, radiotherapy, or systemic chemotherapy, depending on the patient’s performance status, genetic test results, and previous treatment regimens.

Recurrence-free survival (RFS) was defined as the interval from the date of surgery to the date of recurrence. Overall survival (OS) was defined as the time from surgery to death from any cause. Patients were followed until death or the end of the study period (February 28, 2026). Patients who interrupted follow-up or remained under follow-up were recognized as censored; RFS and OS were calculated based on the number of days until censoring.

### 2.5. Subgroup analysis

To evaluate the impact of perioperative shift in PNI on recurrence and prognosis, a subgroup analysis was performed considering the patient background, era of surgery, surgical outcomes, neoadjuvant or adjuvant chemotherapy, and the status of liver metastases, including their number and size. We divided the era of surgery into 2 groups, using the midpoint of the survey period as the boundary. Additionally, because the timing of postoperative PNI measurements differed from that reported in previous studies,^[9,10]^ we conducted a validation study to investigate potential biases related to the measurement dates. We separately validated the results of measurements taken at 3 to 5 weeks postoperatively, aligning with the methodology of a previous study,^[9,10]^ and those taken at 6 and 7 weeks postoperatively.

### 2.6. Comparison of the predictive accuracy of preoperative and perioperative shift in PNI for recurrence and prognosis

We evaluated the utility of preoperative versus perioperative shift in PNI for predicting recurrence and prognosis. The preoperative PNI was assessed using 2 cutoff values: 45, as previously reported,^[11]^ and 48.96, determined by the receiver operating characteristic (ROC) curve analysis for survival (Fig. S1A, Supplemental Digital Content 1). Similarly, the postoperative PNI was evaluated using 2 cutoff values: 46.64, determined by the ROC analysis for survival, and 45 (Fig. S1B, Supplemental Digital Content 1).

### 2.7. Statistical analyses

Statistical analyses were performed using JMP 16 (SAS Institute Inc.). Continuous variables are presented as medians with ranges and were compared using the Mann–Whitney *U*-test. Categorical variables are expressed as counts and percentages and were analyzed using Fisher exact test. RFS and OS were estimated using the Kaplan–Meier method and compared using the log-rank test. Multivariate analysis of survival outcomes was conducted using the Cox proportional hazards regression models. The preoperative PNI cutoff value for survival was determined using ROC analysis. The Cox proportional hazard models were used to estimate the hazard ratio (HR) for RFS and OS for each model. The Akaike information criterion (AIC) was computed for all 5 models: preoperative PNI with a cutoff value of 45, preoperative PNI with a cutoff value of 48.96, postoperative PNI with a cutoff value of 45, postoperative PNI with a cutoff value of 46.64, and perioperative shift in PNI. Among the 5 models, the 1 with the lowest AIC value was the best. Statistical significance was set at *P* < .05.

## 3. Results

### 3.1. Clinical characteristics

The participants’ characteristics are listed in Table 1. The median observation period was 3.3 years. The patients’ backgrounds were similar between the 2 groups, although laparoscopy was more common in the high group (*P* = .065). There were no differences in the surgical outcomes or the rate of patients who received neoadjuvant or adjuvant chemotherapy between the 2 groups.

**Table 1:** Clinical characteristics.

	High group n = 40	Low group n = 68	*P* value
Age, yr, median (range)	62 (41–81)	68 (35–84)	.107
Sex (%)			.281
Male	25 (62.5)	50 (73.5)	
Female	15 (37.5)	18 (26.5)	
Location of tumor (%)			.319
Colon	24 (60.0)	33 (48.5)	
Rectum	16 (40.0)	35 (51.5)	
*T* factor			.581
*T*1	0 (0)	3 (4.4)	
*T*2	4 (10.0)	6 (8.8)	
*T*3	28 (70.0)	44 (64.7)	
*T*4	8 (20.0)	15 (22.1)	
Lymph node metastases			1.000
Yes	27 (67.5)	47 (69.1)	
No	13 (32.5)	21 (30.9)	
Timing of liver metastases			1.000
Synchronous	27 (67.5)	47 (69.1)	
Metachronous	13 (32.5)	21 (30.9)	
Tumor number			.113
Solitary	27 (67.5)	35 (51.5)	
Multiple	13 (32.5)	33 (48.5)	
Maximum tumor size, mm	19.5 (9–80)	22.8 (3.8–80)	.201
Preoperative BMI, kg/m^2^, median (range)	21.7 (15.4–32.0)	21.8 (16.0–33.77)	.602
ASA-PS			.529
3	0 (0)	2 (2.9)	
1, 2	40 (100.0)	66 (97.1)	
Tumor marker			
CEA, ng/mL	5.2 (0.3–58.1)	6.7 (0.5–373)	.523
CA19-9, U/mL	11.0 (1–116)	14.5 (1–1177)	.145
Neoadjuvant chemotherapy			.687
Yes	15 (37.5)	29 (42.6)	
No	25 (62.5)	39 (57.4)	
Surgical approach (%)			.065
Laparoscopy	11 (27.5)	8 (11.8)	
Open*	29 (72.5)	60 (88.2)	
Operation time, min	264.5 (145–512)	283 (103–476)	.275
Blood loss, mL	298 (5–1589)	368 (5–5431)	.472
Blood transfusion (RBC)			1.000
Yes	4	7	
No	36	61	
Hepatectomy			.629
Major	7 (17.5)	15 (22.1)	
Minor	33 (82.5)	53 (77.9)	
Histologic surgical margin			1.000
*R*0	37 (92.5)	62 (91.2)	
*R*1	3 (7.5)	6 (8.8)	
Complications, C–D grade ≥ 2, (%)			
All complications	8 (20.0)	23 (33.8)	.186
Infection complications	6 (15.0)	18 (26.5)	.231
Others	2 (5.0)	5 (7.4)	1.000
Adjuvant chemotherapy			.642
Yes	11 (27.5)	15 (22.1)	
No	29 (72.5)	53 (77.9)	
Recurrence (%)			
All recurrence	12 (30.0)	48 (70.6)	.001
Liver metastasis	9 (22.5)	28 (41.2)	.060
Lung metastasis	2 (5.0)	11 (16.2)	.126
Others	1 (2.5)	9 (13.2)	.088
Death			
All death	7 (17.5)	37 (54.4)	.001
Death related to colorectal cancer	6 (15.0)	37 (54.4)	.001

ASA-PS = American Society of Anesthesiologists Physical Status, BMI = body mass index, CA19-9, CEA, C–D grade = Clavien–Dindo grade, RBC = red blood cell.

* Includes conversion from laparoscopic to open surgery.

### 3.2. Impact of perioperative shift in PNI levels on recurrence and survival

**Figure 2.**
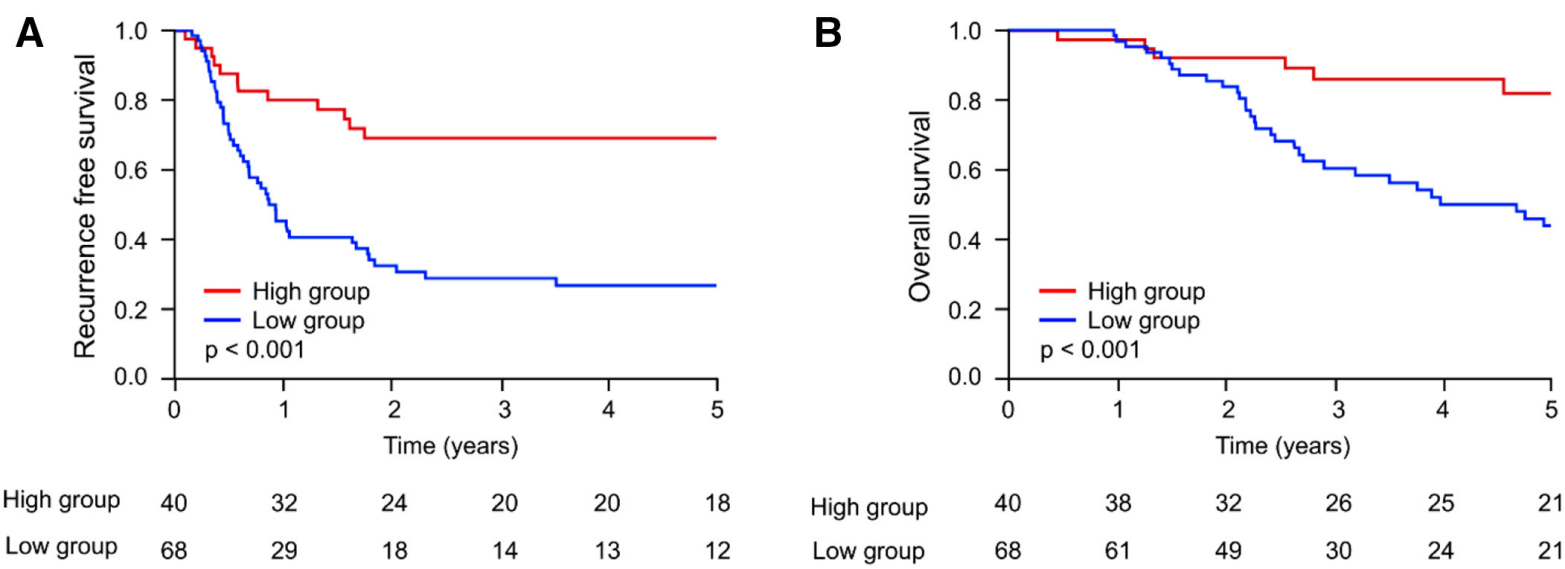
Comparison of overall survival and recurrence-free survival in the Kaplan–Meier analysis. (A) recurrence-free survival, (B) overall survival.

The incidence of recurrence, including both hepatic and pulmonary metastases, was significantly higher in the low group. Additionally, the number of deaths during the observation period was significantly greater in the low group (Table 1). The Kaplan–Meier analysis revealed that both OS and RFS were significantly poorer in the low group compared to the high group (*P* < .001 for both; Fig. 2).

### 3.3. Subgroup analysis

**Figure 3.**
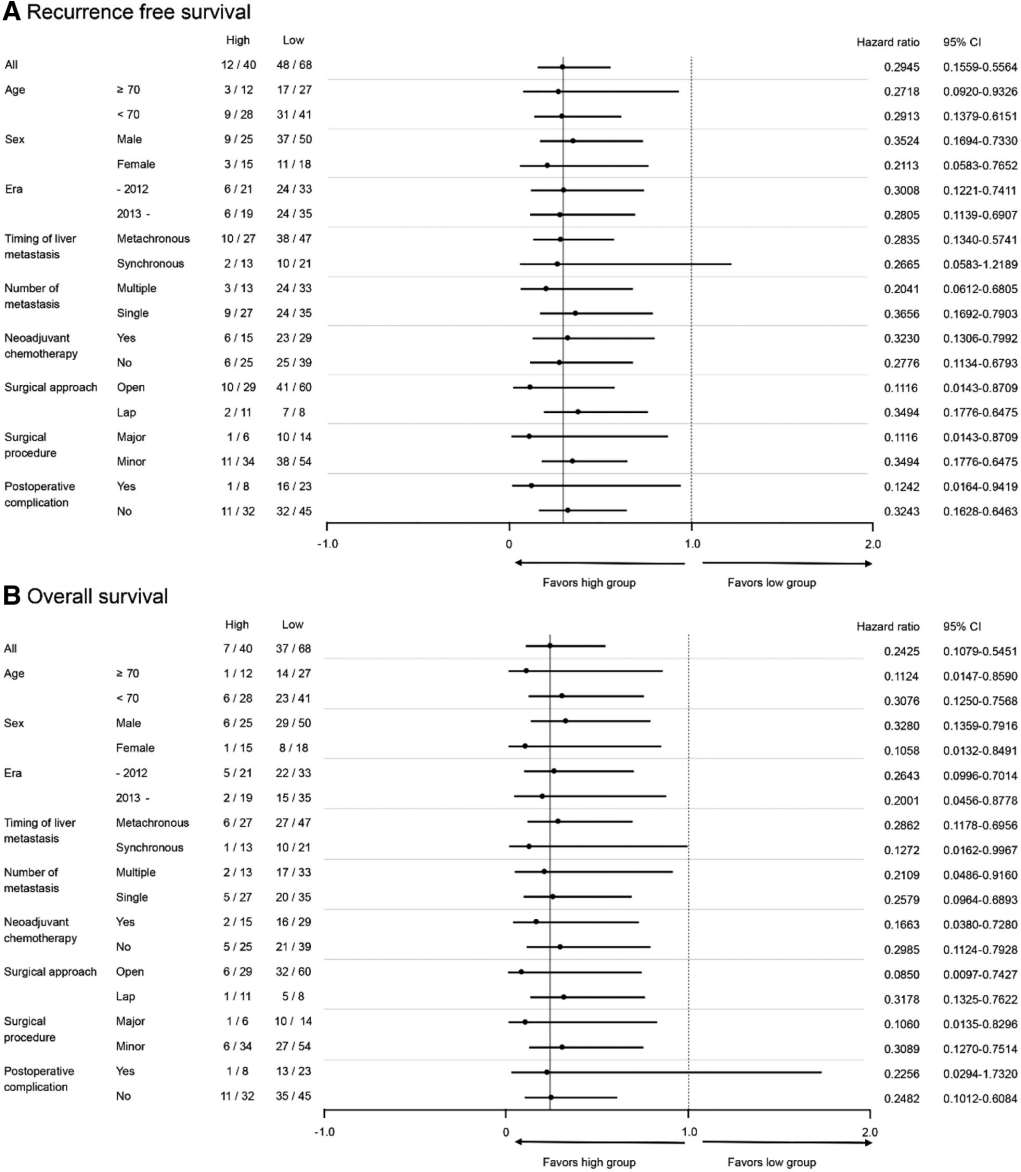
Forest plot of (A) recurrence-free survival and (B) overall survival. CI = confidence interval.

To thoroughly investigate the prognostic impact of perioperative shift in PNI, subgroup analyses were conducted for both OS and RFS. These analyses focused separately on patient background, era of surgery, surgical outcomes, neoadjuvant or adjuvant chemotherapy, and the status of liver metastases, including their number and size. Across all subgroups, except for the synchronous CRLM group, patients in the high group exhibited significantly better RFS outcomes (Fig. 3A); the synchronous CRLM group tended to show better RFS in the high group. Furthermore, the high group had significantly better OS in all subgroups except the postoperative complication group, which tended to show better OS in the high group (Fig. 3B). In addition, no bias was observed due to the measurement dates, and significantly better RFS and OS rates were noted in the high group (Fig. S2, Supplemental Digital Content 1).

### 3.4. Risk factors of recurrence and prognosis after hepatectomy for CRLM

In the multivariate analysis, a perioperative shift in PNI (low group) (HR, 3.788; 95% confidence interval [CI], 2.030–7.621; *P* < .001) and synchronous liver metastasis (HR, 3.338; 95% CI, 1.715–6.964; *P* < .001) were independent predictors of worse RFS (Table 2). Similar results were observed for OS (HR, 4.484; 95% CI, 1.945–10.334; HR, 2.529; 95% CI, 1.183–5.407, respectively) (Table 3).

**Table 2:** Multivariate analysis of the factors affecting recurrence-free survival.

	Multivariate analysis		
	HR	95% CI	*P* value
Age, ≥ 70	1.342	0.681–2.621	.393
Sex, men	1.547	0.847–2.988	.159
Perioperative PNI, low group	3.788	2.030–7.621	<.001
Timing of liver metastases, synchronous	3.338	1.715–6.964	<.001
Tumor number, multiple	0.801	0.448–1.418	.448
Maximum tumor size, > 50 mm	1.153	0.321–3.251	.805
Complications, C–D grade ≥ 2	0.744	0.388–1.355	.350
Neoadjuvant chemotherapy, yes	1.456	0.788–2.709	.231
Adjuvant chemotherapy, yes	0.898	0.406–1.824	.778

C–D grade = Clavien–Dindo grade, CI = confidence interval, HR = hazard ratio, PNI = prognostic nutritional index.

**Table 3:** Multivariate analyses showing factors affecting the overall survival.

	Multivariate analysis		
	HR	95% CI	*P* value
Age, ≥ 70	1.453	0.681–3.100	.334
Sex, men	1.456	0.675–3.141	.338
Perioperative PNI, low group	4.484	1.945–10.334	<.001
Timing of liver metastases, synchronous	2.529	1.183–5.407	.017
Tumor number, multiple	0.638	0.332–1.226	.177
Maximum tumor size, > 50 mm	2.183	0.673–7.076	.193
Complications, C–D grade ≥ 2	0.937	0.474–1.852	.852
Neoadjuvant chemotherapy, yes	0.909	0.446–1.854	.793
Adjuvant chemotherapy, yes	1.270	0.538–2.998	0.586

C–D grade = Clavien–Dindo grade, CI = confidence interval, HR = hazard ratio, PNI = prognostic nutritional index.

### 3.5. Comparison of the predictive accuracy of preoperative, postoperative, and perioperative shift in PNI for recurrence and prognosis

Both preoperative, postoperative, and perioperative shifts in PNI significantly predicted recurrence and prognosis. The AIC analysis indicated that the method utilizing perioperative shift in PNI was superior in predicting recurrence and prognosis (Table 4).

**Table 4:** Predictive accuracy of preoperative, postoperative, and perioperative shift in PNI for recurrence and prognosis using AIC.

Recurrence	AIC	HR	95% CI
Preoperative PNI with a cutoff value of 45	508.57	0.546	0.327–0.911
Preoperative PNI with a cutoff value of 48.96	510.57	0.574	0.310–1.063
Postoperative PNI with a cutoff value of 45	508.57	0.546	0.327–0.911
Postoperative PNI with a cutoff value of 46.64	507.72	0.511	0.296–0.881
Perioperative shift in PNI	496.66	0.295	0.156–0.556
Survival			
Preoperative PNI with a cutoff value of 45	355.59	0.385	0.204–0.726
Preoperative PNI with a cutoff value of 48.96	358.08	0.375	0.167–0.842
Postoperative PNI with a cutoff value of 45	355.59	0.385	0.204–0.726
Postoperative PNI with a cutoff value of 46.64	354.93	0.348	0.171–0.704
Perioperative shift in PNI	349.47	0.243	0.108–0.545

AIC = Akaike information criterion, CI = confidence interval, HR = hazard ratio, PNI = prognostic nutritional index.

## 4. Discussion

This retrospective study investigated the prognostic significance of a perioperative shift in PNI for patients undergoing hepatectomy for CRLM. The results demonstrated that patients classified in the low group based on a perioperative shift in PNI had significantly poorer OS and RFS. These perioperative changes were assessed by comparing preoperative PNI with PNI measured approximately 1 month postoperatively. This enables a simple and practical evaluation without the need for cutoff values or complex stratification methods. Notably, the predictive value of a perioperative shift in PNI was consistent across subgroups defined by patient background, surgical era, operative outcomes, chemotherapy status (neoadjuvant or adjuvant), and characteristics of liver metastases. To our knowledge, this is the 1st study to thoroughly investigate the prognostic relevance of perioperative PNI dynamics in the context of hepatectomy for CRLM. Furthermore, our findings suggest that perioperative shifts in PNI serve as a more precise prognostic indicator than either preoperative or postoperative PNI alone.

Nutrition plays an important role in recovery after gastroenterological surgery.^[16]^ A recent ESPEN practical guideline recommends preoperative nutritional assessment and interventions for patients with severe nutritional risk.^[17]^ Notably, as a preoperative nutritional assessment, the PNI is a well-known biomarker for recurrence and prognosis in many cancer types.^[18]^ However, this study indicated that a perioperative shift in PNI, that is, the change in PNI levels between the preoperative and postoperative periods, more accurately predicted recurrence and prognosis than either preoperative or postoperative PNI alone. This result suggests that an assessment of perioperative recovery in addition to nutritional status may be beneficial for accurate prediction of recurrence and prognosis.

Surgery-induced stress involves systemic effects, such as inflammation, ischemia-reperfusion injury, sympathetic nervous system activation, and increased cytokine release, which together significantly increase the risk of cancer recurrence.^[19]^ Surgical stress is associated with accelerated protein turnover, marked by upregulated protein synthesis and degradation. Typical examples include changes in the concentration of plasma albumin levels; net hepatic synthesis, degradation, and distribution into extravascular compartments due to transcapillary escape dictate the plasma albumin concentration.^[20]^ Therefore, postoperative plasma albumin levels reflect pharmacological characteristics such as antioxidant or transporter status, postoperative inflammatory status, and nutritional state.^[21]^ A recent retrospective study on surgery-induced stress in liver resection showed that changes in plasma albumin levels reflect surgery-induced stress.^[22]^ In addition, the recovery of immune functions, such as lymphocyte levels, reflects recovery from surgical invasion, and a lack of lymphocyte recovery predicts poor prognosis.^[23]^ Therefore, the assessment of PNI changes during the perioperative period would reflect the degree of recovery from surgery.

Recent advancements in minimally invasive surgery and perioperative management have reduced surgically induced stress.^[24–27]^ We believe that laparoscopic surgery and minor hepatectomy might not clearly predict recurrence and prognosis based on perioperative shifts in PNI because of generally lower surgically induced stress compared with those of open surgery and major hepatectomy. However, our subgroup analysis demonstrated that perioperative shifts in PNI could predict recurrence and prognosis, regardless of the surgical approach and extent of liver resection. These findings suggest that perioperative shifts in PNI serve as a robust prognostic indicator even in the context of minimally invasive procedures. Further advancements in minimally invasive surgical techniques, including robotic-assisted surgery and perioperative nutritional management, are essential to further accelerate postoperative recovery.

This study has several limitations. First, it was a retrospective analysis conducted at a single institution. However, the inclusion of consecutive patients helped to minimize selection bias. Second, the relatively long study period may have introduced variability in treatment strategies, including advancements in surgical techniques, changes in neoadjuvant and adjuvant chemotherapy regimens, and improvements in perioperative management. Nevertheless, subgroup analyses stratified by surgical era (up to 2012 vs 2013 onward) demonstrated consistent results, suggesting that the potential bias introduced by temporal changes was limited.

## 5. Conclusions

In conclusion, perioperative shifts in PNI are a significant predictor of recurrence and prognosis after hepatectomy for CRLM, providing a tool for a more precise evaluation than using preoperative or postoperative PNI alone.

## Acknowledgments

The authors would like to express their gratitude to all the patients and medical staff at the institution who contributed to this study.

## Author contributions

**Conceptualization:** Kyota Tatsuta, Mayu Sakata.

**Data curation:** Yuya Iwase, Toru Takagi, Akio Matsumoto, Shinya Ida, Kosuke Sugiyama, Kakeru Torii, Satoru Furuhashi.

**Formal analysis:** Kyota Tatsuta, Mikihiro Shimizu.

**Investigation:** Tadahiro Kojima, Toshiya Akai

**Methodology:** Kyota Tatsuta.

**Project administration:** Mayu Sakata.

**Software:** Kyota Tatsuta, Mikihiro Shimizu.

**Supervision:** Hiroya Takeuchi.

**Validation:** Mikihiro Shimizu.

**Visualization:** Kyota Tatsuta.

**Writing – original draft:** Kyota Tatsuta.

**Writing – review & editing:** Makoto Takeda, Yoshifumi Morita, Hirotoshi Kikuchi, Yoshihiro Hiramatsu, Kiyotaka Kurachi


