# Supplementary material for: Perioperative dynamics of the prognostic nutritional index predict recurrence and survival after resection for colorectal liver metastases
Source: Medicine (Baltimore). 2026 Jul 17;105(29):e49748. doi: 10.1097/MD.0000000000049748 (PMC13384622; doi:10.1097/MD.0000000000049748)
Supplement: Supplementary file 1 [file medi-105-e49748-s001.pdf]

Supplemental Digital Content

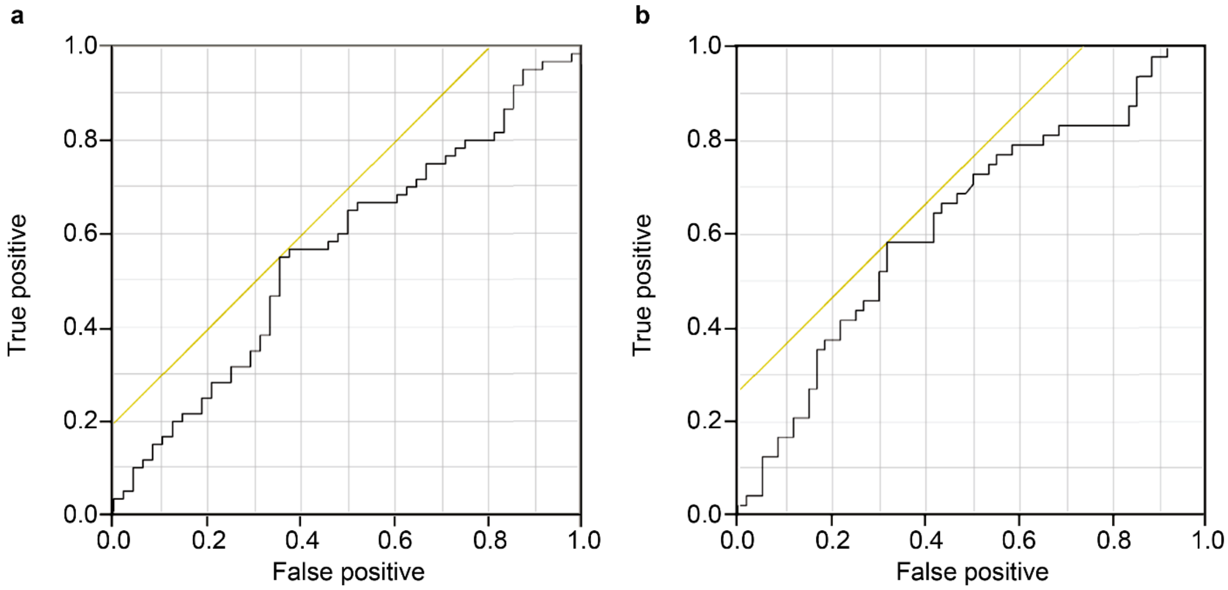

Figure S1. The ROC analysis of preoperative and postoperative PNI predicting mortality

a: The cutoff value of preoperative PNI was 48.96 (sensitivity 0.55, specificity 0.65, AUC 0.57).
b: The cutoff value of postoperative PNI was 46.64 (sensitivity 0.58, specificity 0.68, AUC 0.63)
